# Vaginal microbiota and circulating interferon-stimulated genes in lactating dairy cows at and following time of artificial insemination

**DOI:** 10.1093/jas/skaf399

**Published:** 2025-11-18

**Authors:** Dallas R Soffa, Kyle J Hickman, Joe W Cain, Jennifer A Spencer, Rebecca K Poole

**Affiliations:** Department of Animal Science, Texas A&M University, College Station, TX 77843; Department of Animal Science, Texas A&M University, College Station, TX 77843; Department of Veterinary Integrative Biosciences, Texas A&M University, College Station, TX 77843; Texas A&M AgriLife Research and Extension Center, Texas A&M AgriLife, Stephenville, TX 76401; Department of Animal Science, Texas A&M University, College Station, TX 77843

**Keywords:** bacterial abundance, early gestation, Holstein, myxovirus resistance 2, reproductive microbiome

## Abstract

Reproductive tract microbiota has been shown to shift during early gestation in cattle. However, characterization of the vaginal microbiota during the establishment of pregnancy in lactating dairy cows has yet to be fully determined. Therefore, the objectives of this study were to characterize shifts in the vaginal microbiota from time of artificial insemination (AI) to maternal recognition of pregnancy and analyze its relationship with interferon-stimulated genes (ISGs) in lactating dairy cows. Vaginal swab samples for microbiota analysis and blood were collected from lactating Holstein dairy cows (*n* = 40) on day 0 (d0; time of AI) and day 18 (d18; post-AI during the time of maternal recognition of pregnancy). Pregnancy status was determined via transrectal ultrasonography on d32 (Open, *n* = 18 and Pregnant, *n* = 22). Microbiota analysis for phylum and genus taxonomic classification, as well as α- and β-diversity, was conducted targeting the V4 hypervariable region of the 16S rRNA gene on d0 and d18. RT-qPCR analysis was conducted to analyze expression of ISGs, interferon-stimulated gene 15 kDa (ISG15) and myxovirus resistance 2 (MX2), on d0 and d18. Phyla and genera greater than 1% relative abundance did not differ between Open and Pregnant cows (*P *> 0.10). Shifts in certain bacterial phyla and genera between d0 and d18 (*P *< 0.05), along with lower α-diversity matrices of Simpson’s diversity index (*P *= 0.02) and Shannon’s diversity index (*P *= 0.009) on d18, indicate that time-dependent shifts in bacterial communities may alter reproductive success. This is further supported by the difference between days for the weighted UniFrac β-diversity matrix (*P *= 0.005). Reduced MX2 expression on d18 (*P *= 0.002), and its correlation with Fusobacteria (*r *=* −*0.35; *P *= 0.09), may be indicative of pregnancy failure and thus result in Open cows. These results indicate that shifts in microbiota from day of AI to d18 may influence successful establishment of pregnancy in lactating dairy cows.

## Teaser

Vaginal bacterial communities shift in composition following artificial insemination but are not directly associated with pregnancy status. Rather, correlations of bacteria with interferon-stimulated genes expression indicate that interactions of reproductive microbiota with these signaling molecules are more indicative of successful establishment of pregnancy compared to reproductive microbiota composition alone.

## Introduction

Pregnancy loss within the first 30 d can detrimentally impact financial outcomes for cattle producers. Reproductive inefficiency has been estimated to cost the cattle industry over $1 billion annually, and over $2,000 per pregnancy loss in dairy cattle specifically (Bellows et al., 2002; Lee and Kim, 2007). The majority of pregnancy loss occurs during the early embryonic period, which encompasses these first 30 d of gestation (Inskeep and Dailey, 2005; Reese et al., 2020). Amidst this timeframe, maternal recognition of pregnancy (MRP) occurs between days 15 and 17. This process includes the conceptus-derived interferon tau (IFNτ) signal that will alert the dam of its presence, thereby increasing interferon-stimulated genes (ISGs) expression within the uterus, preventing lysis of the corpus luteum, and aiding in the foundation of pregnancy (Thatcher et al., 1984; Bazer et al., 1997). However, the process of pregnancy establishment may not appear to solely rely on the signaling capabilities of IFNτ with newer evidence that considers the potential role of the reproductive tract microbiota on pregnancy status.

In humans, the reproductive tract microbiota has been shown to impact fertility outcomes based on bacterial abundance and composition. Shifts away from *Lactobacillus*-dominant vaginal microbiota appear to lead to dysbiosis and associated pregnancy loss in most women (Hickey et al., 2012; Huang et al., 2014; Miller et al., 2016). Changes in circulating concentrations of reproductive hormones (that is, estradiol [E2] and progesterone [P4]) during the menstrual cycle and pregnancy appear to shift the microbiome toward more beneficial (that is, *Lactobacillus*) or pathogenic (that is, *Prevotella*) bacteria (Nunn and Forney, 2016; Balle et al., 2020; Saraf et al., 2021). These shifts in the reproductive microbiome have also been noted throughout estrous synchronization protocols and the estrous cycle with changes in reproductive hormone profiles in cattle (Ault et al., 2019; Quereda et al., 2020; Poole et al., 2023a). However, research has found that the reproductive tract microbiota in cattle is not predominantly *Lactobacillus*-driven, and rather the presence of various bacterial genera potentially dependent on reproductive status of the animal (Swartz et al., 2014; Clemmons et al., 2017).

Interest in the common microbial profiles of reproductive tissues in cattle has grown over recent years and surged within the scientific community as a means to evaluate reproductive efficiency. Increased levels of P4 and ISGs (that is, interferon-stimulated gene 15 kDa [ISG15] and myxovirus resistance gene 2 [MX2]) are common indicators for pregnancy establishment in cattle (Skemesh et al., 1973; Gifford et al., 2007; de Melo et al., 2020). Notably, markers such as P4 may be associated with the reproductive tract microbiota to thereby influence pregnancy success or failure (Poole et al., 2023a, 2023b).

Thus, the reproductive microbial populations associated with successful pregnancy establishment in cattle still needs to be determined. The objectives of this study were to characterize the vaginal microbiota at the time of artificial insemination (AI) and post-artificial insemination, during the MRP period, and analyze its relationship with d32 pregnancy status and ISGs expression in lactating dairy cows. It was hypothesized that vaginal microbiota abundance and diversity would differ at time of AI and following time of AI in lactating dairy cows that established pregnancy compared to those that did not become pregnant, and that ISGs expression would be positively correlated with bacterial abundance associated with d32 pregnancy determination.

## Materials and Methods

This study was conducted at the DBA Southland Dairy location of Blue Sky Farms, LLC in Dublin, Texas in November and December 2022. All procedures were approved and carried out in accordance with the Texas A&M AgriLife Institutional Animal Care and Use Committee (#2022-0082).

### Experimental design and sample collection

Multiparous, lactating Holstein dairy cows (*n* = 40) that did not exhibit any clinical or reproductive-related issues, as determined through daily visual observation completed solely by on-farm staff, were housed across pens at the DBA Southland Dairy operation for the duration of the study. Following a 75 d minimum involuntary waiting period, cows were bred to first service AI on d0 following a Double OvSynch synchronization protocol. Sterile swabs were utilized to collect vaginal microbiota samples, in duplicate, on d0 and d18. Day 18 serves as the post-AI sampling timepoint during the MRP period. Using a previously published method for collecting samples, the peritoneal region was wiped using clean paper towels to remove fecal contamination. The vulva was opened, and the swab inserted 6 inches into the vagina where it was then rotated 8 times along the vaginal wall (Smith et al., 2024). Swabs were removed from the vagina and immediately stored in individual, sterile microcentrifuge tubes on ice for 4 hours prior to storage at *−*80 °C for subsequent analysis. Blood was collected via coccygeal venipuncture using K_2_ EDTA collection tubes (BD Vacutainer, Becton, Dickinson and Company, NJ) on d0 and d18. Blood collections were immediately placed on ice and centrifuged at 2500 × *g* and 4 °C for 20 minutes. Plasma was collected, and samples stored at *−*20 °C for P4 analysis. Buffy coats were also collected, and samples were stored at *−*80 °C for real-time quantitative polymerase chain reaction (RT-qPCR) analysis. Milk data provided by the farm was utilized to determine days in milk (DIM) at the time of AI. Pregnancy status based on first service was determined via transrectal ultrasonography on d32 post-AI resulting in either Open (*n* = 18) or Pregnant (*n* = 22).

### Progesterone radioimmunoassay

Progesterone concentrations were quantified according to the manufacturer’s instructions using a commercial double-antibody RIA kit (MP Biomedicals, Santa Ana, CA) with an assay sensitivity of 0.2 ng/mL, as previously described (Pohler et al., 2016; Reese et al., 2019). Concentrations were determined using a calculated standard curve, including high (7.2 ng/mL) and low (0 ng/mL) reference samples for quality control. Samples were completed in a single run and the intra-coefficient of variation was 3.12%.

### Microbiota analysis

Microbiota analysis was performed on the d0 and d18 vaginal swab samples as previously described (Smith et al., 2023, 2024). Briefly, all samples were taken to FERA Diagnostics and Biologicals Corp. (College Station, TX) for DNA extraction and 16S rRNA sequencing. Samples were transferred to 96-well plates and DNA extraction was performed according to the manufacturer’s instructions using the Mag-Bind Universal Pathogen 96 Kit (Omega Bio-Tek, Norcross, GA). The 16S amplicons were amplified by PCR for individual metagenomic DNA samples as previously described (Bicalho et al., 2017b). The V4 hypervariable region of the bacterial 16S rRNA ­bacterial genome was amplified with 515F (5′-GTGCCAGCMGCCGCGGTAA-3′) and 806R (5′-GGACTACHVGGGTWTCTAAT-3′) primers using methods for the Illumina MiSeq platform (Caporaso et al., 2012). Data files have been placed in the Texas A&M University data repository within the Texas Data Repository: https://dataverse.tdl.org/dataverse/dairy_vaginal_microbiome. Resulting sequence reads were utilized for bioinformatics analysis.

### Bioinformatics analysis

Sequence reads were processed for taxon analysis and quality plots assessed utilizing the qiime2 pipeline (https://qiime2.org/; Bolyen et al., 2019) on the Grace server provided by Texas A&M High Performance Research Computing. Low-quality sequences were subsequently removed using DADA2 (Callahan et al., 2016) with a truncation length of 294. For taxonomic classification, the Greengenes 13_8 database with 99% operational taxonomic units (OTUs) pre-trained classifiers for 16S rRNA was used (https://data.qiime2.org/2023.9/common/gg-13-8-99-515-806-nb-classifier.qza). Phylogenetic trees were built with ‘FastTree’ (Price et al., 2010) and ‘mafft’ (Katoh et al., 2002) methods in the qiime2 pipeline, and the resulting BIOM file was exported with metadata and taxonomy information added for further phylogeny metrics.

The α-diversity, within-sample bacterial diversity measurements, and β-diversity, between-sample bacterial diversity measurements, matrices were computed. Alpha diversity matrices included observed OTUs, Chao1, Shannon’s diversity index, and Simpson’s diversity index as measures for bacterial community richness and/or evenness. Beta diversity matrices included unweighted and weighted unique fraction (UniFrac) distance (Weinroth et al., 2022). All plots were generated using R packages ‘phyloseq’ (McMurdie and Holmes, 2013) and ‘ggplot2’ (Wickham and Wickham, 2016).

### RNA extraction and cDNA synthesis

For representative identification of circulating ISGs, buffy coat total RNA was extracted by a modified protocol using TRIzol (Thermo Fisher Scientific, Waltham, USA) reagent along with the Qiagen RNeasy Mini Kit (Qiagen, Hilden, Germany) (da Fonseca et al., 2023). Specifically, buffy coat samples were thawed on ice and 500 μL of TRIzol was added. Sterile micropestles (Eppendorf, Enfield, CT, USA) were used to homogenize the samples. An additional 250 μL of TRIzol was added, and samples were briefly vortexed for 30 seconds. Samples were incubated at room temperature for 3 minutes before 150 μL of chloroform was added, then vortexed for 15 seconds and incubated at room temperature for 3 minutes. Samples were centrifuged at 12,000 × *g* for 15 minutes at 4 °C, and the resulting supernatant was transferred to another tube where an equal volume of 70% ethanol was added to each sample. The resulting solution was added to the columns provided in the Qiagen RNeasy Mini Kit, and RNA was extracted according to the manufacturer’s instructions.

The concentration and quality of RNA samples were determined using a NanoDrop Spectrophotometer (ND-1000; ThermoFisher Scientific). The concentration and purity of total RNA was determined by measuring the ratio of absorbance at 260 to 280 nm. Multiple samples were not used due to low concentration of RNA, resulting in 54 samples (1 sample per cow per collection day; Open, *n* = 13 and Pregnant, *n* = 14) remaining for analysis. To synthesize cDNA, total RNA samples in 10 μL reaction volumes were utilized in the SuperScript II First-Strand Synthesis System (Life Technologies, Carlsbad, USA) according to the manufacturer’s instructions. The cDNA was stored at *−*20 °C until RT-qPCR analysis.

### RT-qPCR

Quantification of specific transcripts were performed by RT-qPCR as previously described (de Melo et al., 2020; Stenhouse et al., 2022). Briefly, the ABI Prism 7900HT system (Applied Biosystems, Foster City, CA, USA) with Power SYBR Green PCR Master Mix (Applied Biosystems) was used to quantify expression of mRNAs encoding for genes of interest. The selected target genes were interferon-stimulated gene 15 kDa (ISG15) and myxovirus resistance gene 2 (MX2). Primers for these genes in cattle have been previously described and are provided in Table 1 (de Melo et al., 2020; Melo et al., 2020). Efficiency and specificity of primers were tested by generating a standard curve from pooled cDNA and the inclusion of a dissociation curve to the RT reaction, respectively. The expression of β-actin (ACTB) and cyclophilin A (PPIA) genes were tested in all samples and used as reference genes. No effects of sample day were detected for the reference genes. All reactions for RT-qPCR were performed at 10 μL volumes in triplicate on a 384-well plate (Life Technologies, Carlsbad, USA). Expression of each gene relative to the expression of the reference genes was normalized by the comparative Ct method corrected for amplification efficiency (Pfaffl, 2001; Melo et al., 2020). Outliers (*n* = 2; values ± 3 SDs) were removed from further analysis.

**Table 1.** Forward and reverse primers sequences of target and reference genes used for quantitative polymerase chain reaction

Gene	GenBank accession no.	Primer sequence (5’ – 3’)
**ISG15**	NM_174366	Forward: GGTATCCGAGCTGAAGCAGTT
		Reverse: ACCTCCCTGCTGTCAAGGT
**MX2**	NM_173941	Forward: CTTCAGAGACGCCTCAGTCG
		Reverse: TGAAGCAGCCAGGAATAGT
**PPIA**	BF230516.1	Forward: GCCATGGAGCGCTTTGG
		Reverse: CCACAGTCAGCAATGGTGATCT
**ACTB**	NM_173979.3	Forward: GGATGAGGCTCAGAGCAAGAGA
		Reverse: TCGTCCCAGTTGGTGACGAT

### Statistical analysis

Progesterone concentrations and DIM were analyzed using PROC GLM in Statistical Analysis Software (SAS; version 9.4, SAS Inst. Inc., Cary, NC) with pregnancy status as a fixed effect and pen as a random effect. Phyla and genera constituting less than 1% relative abundance were removed from further data analysis. Bacterial abundances were non-normally distributed. All bacterial abundance data was analyzed with nonparametric ANOVA in SAS 9.4 with the independent variable of day or pregnancy status. Significance was determined by Wilcoxon exact test for comparisons between days and pregnancy status, and pairwise analysis was conducted using the Dwass-Steel-Critchlow-Fligner (DSCF) test. Alpha diversity matrices within days and d32 pregnancy determination were analyzed with the Wilcoxon rank sum test for pairwise comparisons using R package ‘ggpubr.’ Beta diversity matrices were analyzed with Permutational Multivariate Analysis of Variance Using Distance Matrices (PERMANOVA) using the R function ‘adonis2’ in R package ‘vegan.’ Relative gene expression for day, pregnancy status, and their interaction were analyzed using one-way ANOVA and Tukey’s HSD for pairwise comparisons, respectively, in JMP Pro 17.0. For nonparametric data correlations of bacterial abundances by day and relative gene expression, Spearman’s correlations were analyzed using PROC CORR in SAS 9.4. Correlation results are reported as least square means ± SD. All other results are reported as least square means ± SEM. Statistical significance is reported at *P *≤ 0.05 and tendency is reported at 0.05 < *P *≤ 0.10.

## Results

### Pregnancy rate, progesterone concentration, and DIM

Based on d32 pregnancy determination, 22 cows were pregnant for a 55.0% conception rate. A difference in P4 concentration was observed on d18 between Open and Pregnant cows (*P *= 0.01). Specifically, Open cows displayed lower P4 concentrations on d18 (3.56 ± 0.40 ng/mL) compared to Pregnant cows on d18 (4.82 ± 0.44 ng/mL). There was no difference in DIM between Open (93.87 ± 9.91 DIM) and Pregnant (77.73 ± 11.10 DIM) cows (*P *> 0.10).

### Bacterial abundance—phylum

**Figure 1.**
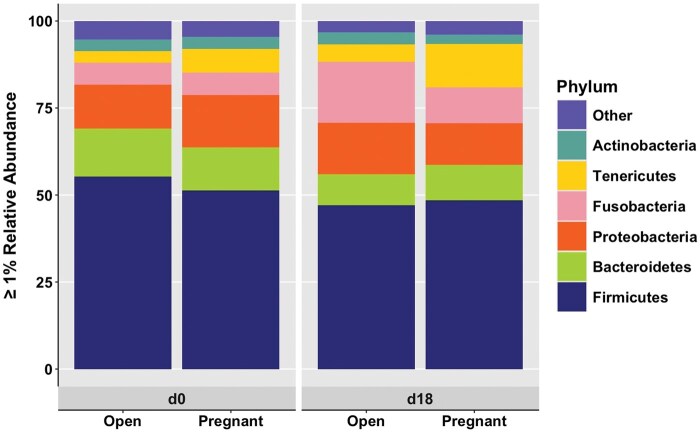
Phyla greater than 1% relative abundance between days (d0 and d18) and pregnancy status (Open and Pregnant). Relative abundance as a percent is shown on the y-axis with pregnancy status within days shown on the x-axis. Differing colors represent different phyla found in greater than 1% relative abundance within the vagina. Phyla less than 1% relative abundance are combined into the “Other” category. Pregnancy status was determined by transrectal ultrasonography on d32 post-artificial insemination.

Phyla greater than 1% relative abundance for Open and Pregnant cows with shifts by day are shown in Figure 1. There were no differences by main effect of pregnancy status (Open vs. Pregnant cows) (*P *> 0.10). There were also no differences in phyla between Open and Pregnant cows on either d0 or d18 (*P *> 0.10). Bacteroidetes (12.96 ± 0.92 vs. 9.64 ± 0.80; *P *= 0.007) had a lower relative abundance on d18 compared to d0. Oppositely, Fusobacteria (6.36 ± 1.35 vs. 13.56 ± 2.76; *P *= 0.02) had a greater relative abundance on d18 compared to d0. More specifically, Bacteroidetes (13.80 ± 1.29 vs. 8.96 ± 1.24; *P *= 0.009) had a greater relative abundance on d0 compared to d18 in Open cows, while Fusobacteria (6.27 ± 2.33 vs. 17.45 ± 4.68; *P *= 0.10) tended to have a lower relative abundance on d0 compared to d18 in Open cows. There were no differences between days in Pregnant cows (*P *> 0.10).

### Bacterial abundance—genus

**Figure 2.**
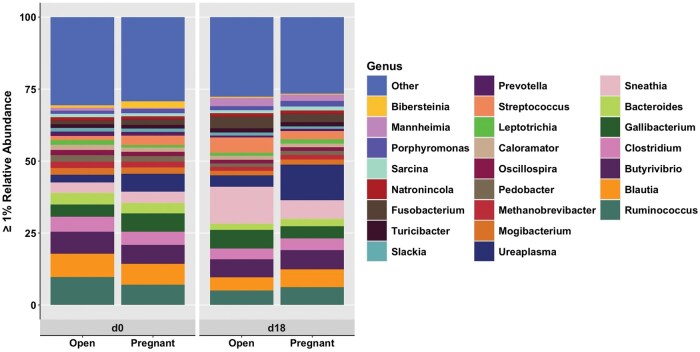
Genera greater than 1% relative abundance between days (d0 and d18) and pregnancy status (Open and Pregnant). Relative abundance as a percent is shown on the y-axis with pregnancy status within days shown on the x-axis. Differing colors represent different genera found in greater than 1% relative abundance within the vagina. Genera less than 1% relative abundance are combined into the “Other” category. Pregnancy status was determined by transrectal ultrasonography on d32 post-artificial insemination.

Bacterial genera greater than 1% relative abundance for Open and Pregnant cows with shifts by day are shown in Figure 2. There were no differences in genera between Open and Pregnant cows (*P *> 0.10). Many genera had greater relative abundance on d0 compared to d18, regardless of pregnancy status, including *Ruminococcus* (8.24 ± 0.65 vs. 5.72 ± 0.65; *P *= 0.008), *Blautia* (7.67 ± 0.51 vs. 5.40 ± 0.57; *P *= 0.001), *Clostridium* (4.83 ± 0.28 vs. 3.87 ± 0.33; *P *= 0.03), *Bacteroides* (3.85 ± 0.41 vs. 2.36 ± 0.30; *P *= 0.005), *Mogibacterium* (2.26 ± 0.19 vs. 1.72 ± 0.21; *P *= 0.04), *Methanobrevibacter* (2.12 ± 0.20 vs. 1.46 ± 0.19; *P *= 0.03), *Pedobacter* (2.05 ± 0.22 vs. 1.27 ± 0.17; *P *= 0.009), *Oscillospira* (1.63 ± 0.11 vs. 1.33 ± 0.14; *P *= 0.05), *Prevotella* (1.35 ± 0.18 vs. 0.66 ± 0.08; *P *= 0.0003), *Slackia* (1.19 ± 0.11 vs. 0.99 ± 0.15; *P *= 0.05), and *Bibersteinia* (1.68 ± 0.93 vs. 0.38 ± 0.15; *P *= 0.01), and *Caloramator* (1.63 ± 0.13 vs. 1.34 ± 0.17; *P *= 0.10) tended to follow the same trend. Oppositely, the genus *Sneathia* (3.80 ± 1.11 vs. 9.37 ± 2.20; *P *= 0.03) had greater relative abundance on d18 compared to d0, and *Leptotrichia* (1.20 ± 0.58 vs. 1.26 ± 0.28; *P *= 0.09) and *Fusobacterium* (1.62 ± 0.55 vs. 3.36 ± 0.92; *P *= 0.08) tended to have greater relative abundance on d18 compared to d0. Furthermore, *Ruminococcus* (9.72 ± 0.86 vs. 7.03 ± 0.89; *P *= 0.04) had greater relative abundance in Open cows compared to Pregnant cows on d0, while *Gallibacterium* (6.50 ± 2.30 vs. 4.30 ± 1.81; *P *= 0.06) tended to be in greater relative abundance in Open cows compared to Pregnant cows on d18. Additionally, *Ruminococcus* (9.72 ± 0.86 vs. 5.06 ± 0.83; *P *= 0.001), *Blautia* (8.11 ± 0.48 vs. 4.58 ± 0.66; *P *= 0.0001), *Clostridium* (5.15 ± 0.34 vs. 3.68 ± 0.46; *P *= 0.02), *Bacteroides* (4.06 ± 0.58 vs. 2.14 ± 0.41; *P *= 0.01), *Methanobrevibacter* (2.25 ± 0.28 vs. 1.31 ± 0.25; *P *= 0.03), *Pedobacter* (2.22 ± 0.32 vs. 1.16 ± 0.23; *P *= 0.02), *Prevotella* (1.42 ± 0.19 vs. 0.56 ± 0.10; *P *= 0.0002), and *Bibersteinia* (0.87 ± 0.44 vs. 0.42 ± 0.30; *P *= 0.03) were greater in relative abundance on d0 compared to d18 in Open cows, and *Oscillospira* (1.80 ± 0.15 vs. 1.31 ± 0.23; *P *= 0.06) and *Slackia* (1.33 ± 0.18 vs. 1.10 ± 0.28; *P *= 0.10) tended to follow the same trend. On the other hand, genera *Sneathia* (3.66 ± 1.69 vs. 12.91 ± 4.04; *P *= 0.10) and *Fusobacterium* (1.25 ± 0.67 vs. 4.07 ± 1.57; *P *= 0.10) tended to be greater in relative abundance on d18 compared to d0 in Open cows. There were no differences between d0 and d18 in Pregnant cows (*P *> 0.10).

### Alpha-diversity

**Figure 3.**
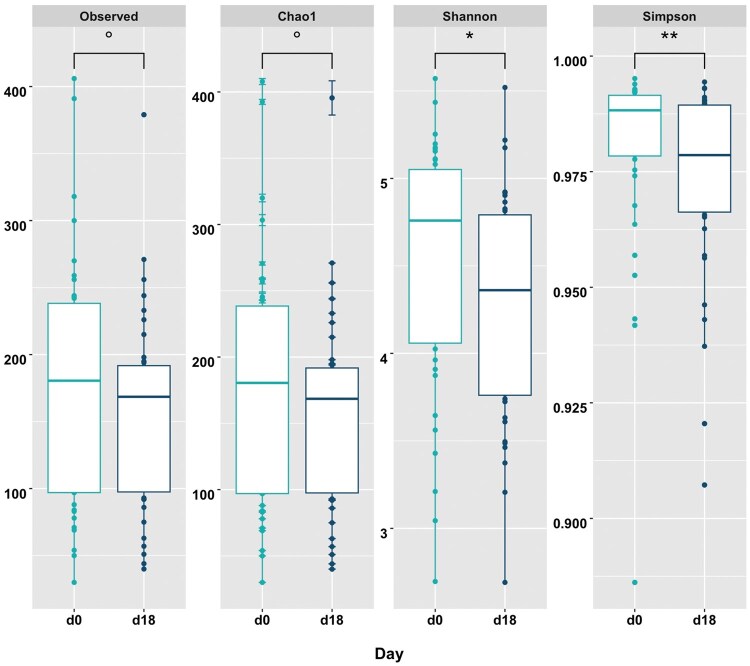
Box plots showcasing α-diversity matrices (Observed [observed OTUs], Chao1, Shannon [Shannon’s diversity index], Simpson [Simpson’s diversity index]) for differences by day. Alpha diversity matrices are denoted along the top, with their corresponding values on the y-axis and days along each bottom x-axis. 0.10 ≥ *P *≥ 0.05; *P ≤ 0.05; **P ≤ 0.01.

**Figure 4.**
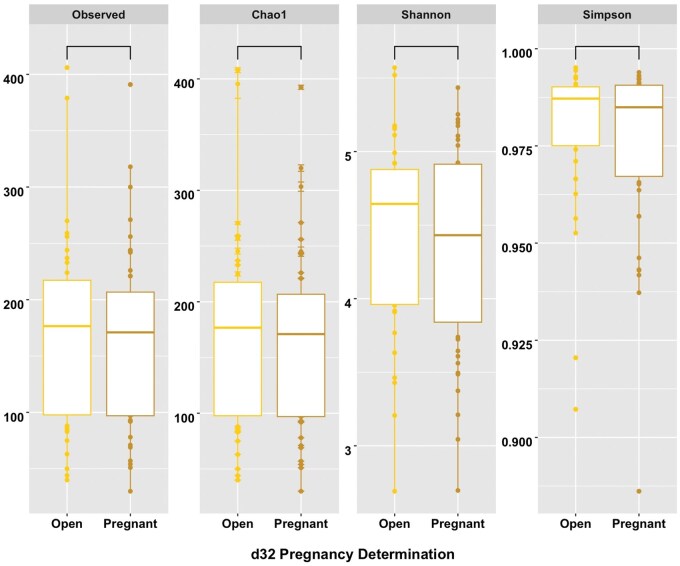
Box plots showcasing α-diversity matrices (Observed [observed OTUs], Chao1, Shannon [Shannon’s diversity index], Simpson [Simpson’s diversity index]) for differences by pregnancy status (Open and Pregnant). Alpha diversity matrices are denoted along the top, with their corresponding values on the y-axis and pregnancy status along each bottom x-axis. Pregnancy status was determined by transrectal ultrasonography on d32 post-artificial insemination.

For α-diversity matrices within days, there was a difference between d0 and d18 for Simpson’s diversity index (*P *= 0.02) and Shannon’s diversity index (*P *= 0.009; Figure 3). There tended to be differences for α-diversity matrices Chao1 (*P *= 0.06) and observed OTUs (*P *= 0.06; Figure 3). Between Open and Pregnant cows, there were no differences for all α-diversity matrices (*P *> 0.10; Figure 4).

### Beta-diversity

**Figure 5.**
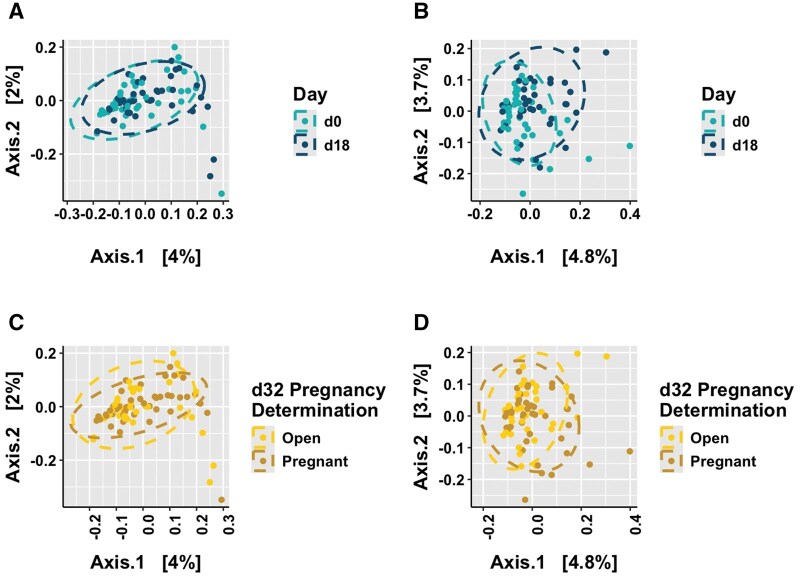
Principal coordinate analyses for β-diversity using unique fraction (UniFrac) matrices. (A) Unweighted UniFrac between days (*P *> 0.10), (B) Weighted UniFrac between days (*P *= 0.005), (C) Unweighted UniFrac between pregnancy status (*P *> 0.10), and (D) Weighted UniFrac between pregnancy status (*P *> 0.10). Ellipses, shown by the dashed circles, designate the 95% CI and each point represents one data point at that specific coordinate. The axes are labeled with the percentage of variation explained. Pregnancy status was determined by transrectal ultrasonography on d32 post-artificial insemination.

There was a difference between days for the weighted UniFrac β-diversity matrix (*P *= 0.005), yet no difference between days for the unweighted UniFrac β-diversity matrix (*P *> 0.10). Additionally, there were no differences between pregnancy status for both weighted and unweighted UniFrac β-diversity matrices (*P *> 0.10; Figure 5).

### Interferon-stimulated genes expression

#### Mean relative mRNA expression

**Figure 6.**
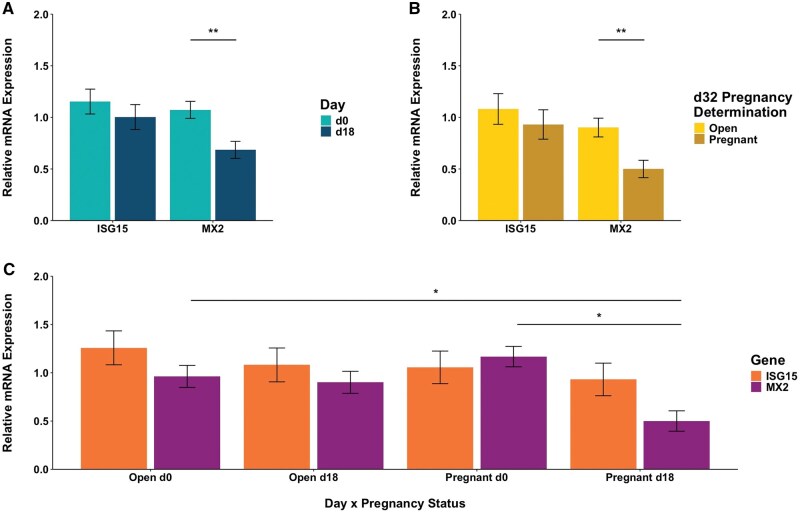
Quantification of relative mRNA expression. (A) ISG15 and MX2 between days with a difference between d0 and d18 for MX2 (*P *= 0.002), (B) ISG15 and MX2 between pregnancy status with a difference in MX2 between Open and Pregnant (*P *= 0.004), (C) ISG15 between days and pregnancy status and MX2 between days and pregnancy status with differences for MX2 between Pregnant d18 and Pregnant d0 (*P *= 0.004) and Pregnant d18 and Open d0 (*P *≤ 0.05). Relative mRNA expression is shown on each y-axis with either day, pregnancy status, or day by pregnancy status shown on the x-axis. Pregnancy status was determined by transrectal ultrasonography on d32 post-artificial insemination. *P ≤ 0.05; **P ≤ 0.01.

Mean relative mRNA expression of ISG15 and MX2 between days are shown in Figure 6A. There was no difference in mean relative mRNA expression of ISG15 between d0 and d18 (*P *> 0.10). There was a difference observed in mean relative mRNA expression of MX2 between d0 and d18 (1.07 ± 0.08 vs. 0.69 ± 0.08, respectively; *P *= 0.002). Mean relative mRNA expression of early pregnancy markers between pregnancy status are shown in Figure 6B. There was no difference in mean relative mRNA expression of ISG15 between Open and Pregnant cows (*P *> 0.10). Mean relative mRNA expression of MX2 was greater in Open versus Pregnant cows (0.90 ± 0.09 vs. 0.50 ± 0.08, respectively; *P *= 0.004). Mean relative mRNA expression of early pregnancy markers between pregnancy status across days are shown in Figure 6C. There were no differences in mean relative mRNA expression of ISG15 between all groups (*P *> 0.10). Mean relative mRNA expression of MX2 was lower in Pregnant cows on d18 (0.50 ± 0.11) versus Open cows on d0 as well as versus Pregnant cows on d0 (0.96 ± 0.11 and 1.17 ± 0.11, respectively; *P *< 0.05).

#### Bacterial abundance correlations—phylum

The mRNA expressions were correlated with phyla abundance data by day, regardless of pregnancy status (Table 2). There were no correlations between the mean relative mRNA expression of MX2 on d0 and phyla relative abundance. On d0, the mean relative mRNA expression of ISG15 was negatively correlated with Tenericutes (*r *=* −*0.43; *P *= 0.03). On d18, the mean relative expression of MX2 tended to be negatively correlated with Fusobacteria (*r *=* −*0.35; *P *= 0.09). There were no significant correlations between the mean relative mRNA expression of ISG15 on d18 with bacterial abundance of phyla.

**Table 2.** Spearman correlations between phyla greater than 1% relative abundance and early pregnancy marker (ISG15 and MX2) relative mRNA expression between days

			Gene			
			ISG15		MX2	
Phylum	Day	Relative abundance^1^	Spearman (*r*)	*P*-value	Spearman (*r*)	*P*-value
**Firmicutes**	d0	54.11 ± 16.16	0.03	0.90	*−*0.14	0.52
	d18	51.10 ± 22.51	0.33	0.11	*−*0.08	0.71
**Bacteroidetes**	d0	13.87 ± 5.61	0.01	0.96	*−*0.006	0.98
	d18	9.55 ± 4.80	*−*0.21	0.31	*−*0.04	0.86
**Proteobacteria**	d0	13.78 ± 12.27	*−*0.02	0.93	0.06	0.78
	d18	11.86 ± 11.65	0.04	0.85	*−*0.09	0.68
**Fusobacteria**	d0	6.79 ± 11.26	0.10	0.65	*−*0.03	0.89
	d18	11.98±16.43	*−*0.35	0.09	0.29	0.16
**Tenericutes**	d0	3.30 ± 4.54	*−*0.43	0.03	0.18	0.39
	d18	8.72 ± 17.61	*−*0.22	0.29	*−*0.04	0.85
**Actinobacteria**	d0	3.30 ± 3.62	*−*0.21	0.32	0.07	0.75
	d18	3.22 ± 1.99	0.26	0.21	*−*0.04	0.85

1 Relative abundance provided as mean ± SD.

#### Bacterial abundance correlations—genus

The mRNA expressions were also correlated with bacterial genera abundance data by day, regardless of pregnancy status, displayed in Table 3. There were no correlations between mean relative mRNA expression of MX2 on d0 and genera relative abundance (*P *> 0.10). The mean relative mRNA expression of ISG15 was negatively correlated with *Ureaplasma* on d0 (*r *=* −*0.41; *P *= 0.04). On d18, *Streptococcus* (*r *= 0.45; *P *= 0.02) was positively correlated with the mean relative expression of MX2 on d18, and *Mogibacterium* (*r *= 0.34; *P *= 0.09) and *Mannheimia* (*r *= 0.38; *P *= 0.06) tended to follow this same trend. Oppositely, *Sneathia* (*r *=* −*0.43; *P *= 0.03) was negatively correlated with the mean relative mRNA expression of MX2 on d18, and *Porphyromonas* (*r *=* −*0.34; *P *= 0.09) tended to be. Interestingly, *Sneathia* (*r *= 0.40; *P *= 0.05) was positively correlated with the mean relative expression of ISG15 on d18.

**Table 3.** Spearman correlations between genera greater than 1% relative abundance and early pregnancy marker (ISG15 and MX2) relative mRNA expression between days

			Gene			
			ISG15		MX2	
Genus	Day	Relative abundance^1^	Spearman (*r*)	*P*-value	Spearman (*r*)	*P*-value
***Ruminococcus***	d0	8.74 ± 4.35	0.11	0.59	*−*0.18	0.38
	d18	6.31 ± 4.21	*−*0.24	0.25	0.22	0.29
***Blautia***	d0	8.03 ± 3.49	*−*0.19	0.36	*−*0.16	0.44
	d18	5.74 ± 3.50	*−*0.25	0.24	0.27	0.20
***Butyrivibrio***	d0	7.15 ± 3.44	*−*0.04	0.85	*−*0.15	0.48
	d18	7.25 ± 5.12	*−*0.14	0.52	0.18	0.40
***Clostridium***	d0	5.04 ± 1.66	0.06	0.78	*−*0.21	0.32
	d18	4.08 ± 2.09	*−*0.05	0.80	0.18	0.40
***Gallibacterium***	d0	6.54 ± 12.36	*−*0.07	0.75	0.19	0.36
	d18	4.18 ± 7.58	0.14	0.51	*−*0.29	0.17
***Bacteroides***	d0	4.31 ± 2.54	0.002	0.99	*−*0.03	0.90
	d18	2.55 ± 1.96	*−*0.29	0.16	0.31	0.13
***Sneathia***	d0	4.42 ± 7.89	0.20	0.34	*−*0.06	0.79
	d18	8.13 ± 12.99	0.40	0.05	*−*0.43	0.03
***Ureaplasma***	d0	2.65 ± 5.16	*−*0.41	0.04	0.20	0.33
	d18	8.10 ± 18.62	*−*0.03	0.88	*−*0.03	0.87
***Mogibacterium***	d0	2.31 ± 1.21	*−*0.06	0.77	*−*0.07	0.74
	d18	1.80 ± 1.31	*−*0.19	0.37	0.34	0.09
***Methanobrevibacter***	d0	2.14 ± 1.22	0.06	0.79	*−*0.15	0.46
	d18	1.60 ± 1.20	*−*0.16	0.44	0.21	0.31
***Pedobacter***	d0	2.27 ± 1.29	0.17	0.42	*−*0.07	0.74
	d18	1.36 ± 1.19	*−*0.31	0.13	0.30	0.15
***Oscillospira***	d0	1.71 ± 0.71	0.08	0.69	*−*0.17	0.41
	d18	1.47 ± 0.96	*−*0.25	0.24	0.25	0.23
***Caloramator***	d0	1.67 ± 0.80	0.19	0.37	*−*0.06	0.78
	d18	1.51 ± 1.15	*−*0.14	0.52	0.32	0.12
***Leptotrichia***	d0	0.75 ± 1.56	0.09	0.66	0.10	0.65
	d18	1.23 ± 1.91	*−*0.04	0.86	*−*0.16	0.45
***Streptococcus***	d0	1.55 ± 2.20	0.006	0.98	0.15	0.49
	d18	4.58 ± 13.99	*−*0.16	0.44	0.45	0.02
***Prevotella***	d0	1.58 ±1.35	*−*0.25	0.23	*−*0.10	0.62
	d18	0.63 ± 0.43	*−*0.27	0.19	0.32	0.12
***Slackia***	d0	1.27 ± 0.72	*−*0.06	0.78	*−*0.08	0.72
	d18	1.10 ± 1.08	*−*0.18	0.39	0.31	0.13
***Turicibacter***	d0	1.19 ± 1.04	*−*0.04	0.84	0.12	0.57
	d18	1.53 ± 1.36	*−*0.17	0.41	0.23	0.28
***Fusobacterium***	d0	1.92 ± 3.86	0.09	0.67	0.10	0.64
	d18	3.07 ± 5.84	0.24	0.24	*−*0.32	0.11
***Natronincola***	d0	1.11 ± 0.68	0.04	0.83	0.06	0.79
	d18	1.30 ± 1.06	*−*0.16	0.44	0.26	0.20
***Sarcina***	d0	1.04 ±0.68	*−*0.01	0.96	0.11	0.61
	d18	1.29 ± 1.24	*−*0.20	0.34	0.17	0.42
***Porphyromonas***	d0	1.33 ± 1.27	0.16	0.46	0.09	0.67
	d18	1.69 ± 2.37	0.22	0.29	*−*0.34	0.09
***Mannheimia***	d0	0.70 ± 2.59	0.05	0.83	*−*0.19	0.37
	d18	2.55 ± 9.50	*−*0.28	0.17	0.38	0.06
***Bibersteinia***	d0	0.36 ± 1.14	0.14	0.49	0.07	0.74
	d18	0.44 ± 1.16	*−*0.21	0.32	0.23	0.27

1 Relative abundance provided as mean ± SD.

## Discussion

Bacterial composition of healthy reproductive tissues continues to be investigated as an indicator of fertility across species. However, characterization of vaginal microbiota during the establishment of pregnancy in lactating dairy cows has yet to be fully elucidated. Therefore, the objectives of this study were to characterize shifts in the vaginal microbiota from the time of AI to post-AI, during the MRP period, and analyze its relationship with ISGs in lactating dairy cows.

The Human Microbiome Project began the vast commitment of research in the role of bacterial abundance and composition on reproductive tissue function (Peterson et al., 2009). In women, the vaginal microbiota has been found to shift throughout both the menstrual cycle and pregnancy (Nunn and Forney, 2016; Balle et al., 2020; Saraf et al., 2021). Previous work has established similar associations in which circulating reproductive hormones (that is, E2 and P4) may play a role in shifting the microbial populations in cattle (Ault et al., 2019; Quereda et al., 2020; Poole et al., 2023a). Similar to these studies, phyla Bacteroidetes, Firmicutes, and Proteobacteria were the most abundant in the current work. Others have also noted Actinobacteria, Fusobacteria and Tenericutes as prominent phyla in vaginal tissues as shown in this study (Chen et al., 2020; Quadros et al., 2020; Moreno et al., 2021). Interestingly, there were no differences in phyla relative abundance or diversity matrices between pregnancy status. Despite this, shifts were observed between days in Open cows indicating that hormone profiles may be a driver of bacterial abundance and composition within the reproductive tract. Previous research has noted that P4 appears to be the dominant hormonal influence on the microbiota. Specifically, high P4 seems to increase bacterial abundance and diversity (Poole et al., 2023a). Due to the lack of differences in bacterial communities between pregnancy status, despite higher P4 profiles in Pregnant cows on d18, P4 may in fact play a smaller role in altering the reproductive microbiota than previously proposed. However, it is important to note the experimental differences in animals between the previous and current study. Poole et al. (2023a) utilized cycling beef heifers, while the current study analyzed data of pregnant, lactating dairy cows. Other studies have noted that circulating P4 concentrations during follicular wave development and time of AI alters pregnancy outcomes differently between beef and dairy cattle (Wiltbank et al., 2014; Stevenson and Lamb, 2016). Progesterone profiles have also been shown to decrease in cyclic cattle by d14 compared to those at d14 in pregnant cattle (Pope et al., 1969). Together, this indicates that breed and reproductive status may alter P4 profiles and subsequently shift bacterial composition within the vagina differently.

This study also noted a greater number of bacterial shifts from d0 to d18 in Open cows compared to a lack of differences between days in Pregnant cows for both phyla and genera. In dairy cattle, the majority of reproductive microbiota research has focused on healthy versus diseased states within the uterus (Bicalho et al., 2017a; Cunha et al., 2018; Miranda-CasoLuengo et al., 2019). Shifts in relative abundances of phyla Bacteroidetes and Fusobacteria have been associated with postpartum reproductive diseases, resulting in increased pregnancy failure (Santos et al., 2011; Bicalho et al., 2017b). In the current study, Bacteroidetes was greater on d0 in Open cows while Fusobacteria was greater on d18 in Open cows. This is notable as these changes may indicate a pathogenic function, where such a shift in microbial abundance may elicit a maternal immune response that then results in cows diagnosed as Open at the d32 pregnancy determination. Interestingly, two of the genera greater than 1% relative abundance that differed within the phylum Bacteroidetes were *Prevotella* and *Pedobacter*, which have been associated with reproductive disease and uterine health in dairy cattle (Sheldon et al., 2004; Jeon et al., 2015; Bicalho et al., 2017b). Furthermore, the combined greater abundance of Bacteroidetes on d0 and Fusobacteria on d18 in Open cows may further coincide with previous work indicating such shifts as pathogenic with increased diseased states in cattle (Jeon et al., 2015; Bicalho et al., 2017b). However, due to the lack of reproductive-related issues as observed solely by on-farm staff, we cannot confirm connections to diseased states. Additionally, phylum Firmicutes did not differ between pregnancy status or day, however, genera within Firmicutes did display shifts. Specifically, *Ruminococcus* had greater abundance on d0 in Open cows compared to Pregnant cows. Along with *Blautia, Clostridium*, and *Oscillospira* within Firmicutes, *Ruminococcus* was also greater in abundance in Open cows on d0 compared to d18. Conversely, *Sneathia*, *Leptotrichia*, and *Fusobacterium* within phylum Fusobacteria were greater in relative abundance on d18 in Open cows compared to d0. Thus, the distribution and/or ratios of certain bacterial phyla and genera may be more indicative of reproductive microbiota associated with early pregnancy failure.

The current study also noted lower α-diversity matrices on d18 compared to d0. Alpha diversity accounts for the number of different bacterial populations within a sample. Both Simpson’s and Shannon’s diversity indices consider the richness and evenness of the samples, while Chao1 and Observed OTUs only evaluate the richness of bacterial populations present (Weinroth et al., 2022). The lower α-diversity on d18 for all alpha matrices suggests disparity in the evenness and richness of bacterial populations, and that increased levels of P4 by d18 may lower bacterial diversity. This is in accordance with findings in women, where increased levels of P4 during pregnancy stabilize bacterial composition and decrease biodiversity (Huang et al., 2014; Nunn and Forney, 2016; Saraf et al., 2021). Additionally, the only difference in β-diversity matrices was between days for weighted UniFrac, which coincides with these results to further indicate the possible role of P4. Weighted UniFrac takes into account both the relative abundance and presence or absence of the microbe, while unweighted UniFrac only evaluates the presence or absence of a microbe (Weinroth et al., 2022). This difference, combined with the α-diversity results and shifts in bacterial genera, suggests that changes in microbiota between day of AI and following time of AI appears to play a larger role in altering reproductive success.

Of further note in the current study was the expression of ISG15 and MX2. Previous work has demonstrated the use of ISG15 and MX2 as markers capable of determining pregnancy during early gestation in cattle. Increased expression of ISG15 in peripheral blood leukocytes between days 18 and 20 in pregnant heifers and cows compared to open females correlates to increased IFNτ secretion by the conceptus (Gifford et al., 2007; de Melo et al., 2020). However, the current study contradicts these findings as ISG15 did not differ between pregnancy status or day. Concurrently, MX2 was lower in Pregnant cows on d18 compared to d0 for both Open and Pregnant females. This is similar to a study where pregnant dairy heifers had an increased expression of MX2 on d0 compared to d18 that was attributed to a potential viral encounter; however, the current study had no immunocompromised cattle based on on-farm personnel checks (Stevenson et al., 2007). Additional studies have also demonstrated changes in reliability of ISGs expression analyses for pregnancy determination, such as differences between parity, species, and day where expression is greater in heifers, beef cattle, and at day 20 post-AI, respectively (Green et al., 2010; Yoshino et al., 2018; de Melo et al., 2020). Lactational status may also alter MX2 expression, thereby increasing gene expression and false positive pregnancy diagnosis (Pugliesi et al., 2014). Together, these results indicate contradictory ISG expression in circulating white blood cells impairs the accuracy of pregnancy diagnosis utilizing the relative expression of these genes. It also demonstrates the continued research needed to determine which ISG is most conducive to utilize as an early pregnancy detection method in cattle during different physiological states such as lactation.

With the observed ISG expressions, it was also imperative to understand the potential association with specific bacterial phyla and genera. Expression of MX2 may be negatively driven by phylum Fusobacteria on d18. This was also reflected at the genera level with greater bacterial relative abundance of *Sneathia* diminishing expression on d18, further indicating it may act pathogenically on pregnancy establishment. This may additionally be supported by the positive correlation between relative abundance of *Sneathia* and ISG15 on d18. Since Fusobacteria has been associated with metritis in dairy cattle previously, ISG15 may be more indicative of maternal immune responses while MX2 may be a better indicator of pregnancy status in early gestation (Bicalho et al., 2017b). Also of note is *Ureaplasma*, under phylum Tenericutes, which has been most commonly associated with the healthy vaginal microbiota in cattle to potentially aid in promotion of pregnancy establishment (Kudo et al., 2021; Moreno et al., 2021). In the current study, both the genus and phylum were negatively correlated with ISG15 on d0. This may indicate a delicate balancing act between microbiota abundance and endometrial ISGs expression on the maternal immune response to prepare the uterine environment for promotion of pregnancy. Meanwhile, *Streptococcus* and *Mogibacterium*, under phylum Firmicutes, and *Mannheimia*, under phylum Proteobacteria, were positively associated with MX2 on d18. Bicalho and others noted that these two phyla are typically associated with healthy and pregnant animals, potentially further insinuating MX2 as a better ISG indicator of pregnancy establishment (Bicalho et al., 2017b). Taken together, cows in the current study may have had increased pathogenic bacteria and diminished MX2 expression on d18 in resulting Open cows.

## Conclusions

Overall, these results indicate that shifts in microbiota from day of AI to when MRP should be occurring may influence successful establishment of pregnancy in lactating dairy cows, supporting our hypothesis that bacterial abundance and diversity will shift at and following time of AI. Specifically, a greater abundance of Fusobacteria and reduced expression of MX2 following time of AI could lead to an unstable reproductive environment incapable of pregnancy establishment. The uncertainty of progesterone’s role in shifting the vaginal microbiota during early gestation warrants further consideration as to whether these bacterial shifts are influenced by hormone concentrations. In addition, with conflicting results across studies, additional research is needed to further elucidate the complex relationship between vaginal microbiota and interferon-stimulated genes during early pregnancy establishment. Understanding the requirements necessary in the establishment of healthy reproductive tract microbiota that are associated with successful pregnancy establishment is essential for the future improvement of reproductive efficiency in cattle.

## Abbreviations:

ACTB: β-actin
AI: artificial insemination
DIM: days in milk
DSCF: Dwass-Steel-Critchlow-Fligner
E2: estradiol
IFNτ: interferon tau
ISG: interferon-stimulated gene
ISG15: interferon-stimulated gene 15 kDa
MRP: maternal recognition of pregnancy
MX2: myxovirus resistance gene 2
OTUs: observed taxonomic units
PERMANOVA: Permutational Multivariate Analysis of Variance Using Distance Matrices
PPIA: cyclophilin A
P4: progesterone
RT-qPCR: real-time quantitative polymerase chain reaction
